# Evaluating Mental Load During Realistic Driving Simulations by Means of Round the Ear Electrodes

**DOI:** 10.3389/fnins.2019.00940

**Published:** 2019-09-04

**Authors:** Edmund Wascher, Stefan Arnau, Julian Elias Reiser, Georg Rudinger, Melanie Karthaus, G. Rinkenauer, F. Dreger, Stephan Getzmann

**Affiliations:** ^1^Leibniz Research Centre for Working Environment and Human Factors (IfADo), TU Dortmund University, Dortmund, Germany; ^2^Society for Empirical Social Research and Evaluation (uzbonn), University of Bonn, Bonn, Germany

**Keywords:** EEG, driving, Alpha power, Theta Power, mental work load

## Abstract

Film based round the ear electrodes (cEEGrids) provide both, the accessibility of unobtrusive mobile EEG as well as a rapid EEG application in stationary settings when extended measurements are not possible. In a large-scale evaluation of driving abilities of older adults (N > 350) in a realistic driving simulation, we evaluated to what extent mental demands can be measured using cEEGrids in a completely unrestricted environment. For a first frequency-based analysis, the driving scenario was subdivided into different street segments with respect to their task loads (low, medium, high) that was *a priori* rated by an expert. Theta activity increased with task load but no change in Alpha power was found. Effects gained clarity after removing pink noise effects, that were potentially high in this data set due to motion artifacts. Theta fraction increased with task load and Alpha fraction decreased. We mapped this effect to specific street segments by applying a track-frequency analysis. Whilst participants drove with constant speed and without high steering wheel activity, Alpha was high and theta low. The reverse was the case in sections that required either high activity or increased attentional allocation to the driving context. When calculating mental demands for different street segments based on EEG, this measure is highly significant correlated with the experts’ rating of task load. Deviances can be explained by specific features within the segments. Thus, modulations in spectral power of the EEG were validly reflected in the cEEGrids data. All findings were in line with the prominent literature in the field. The results clearly demonstrate the usability of this low-density EEG method for application in real-world settings where an increase in ecological validity might outweigh the loss of certain aspects of internal validity.

## Introduction

The recent development of film based round the ear electrodes (cEEGrids) is an important step to access EEG in situations in which the recording of electrophysiological brain activity has previously not been possible. Compared to multichannel Cap-EEG, the application of cEEGrids is significantly less time-consuming, whilst the signal quality is still sufficiently high to derive valid and reliable measures (Bleichner et al., 2016; Bleichner and Debener, 2017; Denk et al., 2018; Sterr et al., 2018). These properties render the cEEGrid electrodes particularly useful when lab-time is limited as well as in mobile real-life situations in which minimizing the interference due to the recording is a concern (Debener et al., 2015; Sterr et al., 2018; Mikkelsen et al., 2019). Especially when measuring EEG in non-stationary or outside-of-the-lab situations, recording technologies need to overcome present restrictions due to conventional electrode setups (Symeonidou et al., 2018). Generally speaking, the specific advantages of the cEEGrid electrodes over multichannel Cap-EEG are especially relevant in situations in which a high ecological validity is desired.

It has repeatedly been shown that the EEG signal derived from cEEGrid electrodes may provide reliable correlates for basic mental processing (Mirkovic et al., 2016; Bleichner and Debener, 2017). Both, auditory and visually evoked potentials were shown to be measurable with these types of electrodes (Bleichner et al., 2016; Pacharra et al., 2017). Moreover, it has been recently shown that even the localization of cortical sources of the signal is possible, since the projections of the electric fields of potentially relevant dipoles are distinguishable by using various combinations of the cEEGrid channels (Bleichner et al., 2016; Bleichner and Debener, 2017). It could also be demonstrated that it is feasible to obtain reliable correlates of cognitive activity in the frequency domain from a signal recorded via cEEGrid electrodes. Systematic and task-related modulations of Alpha activity have been reported when accessed in the laboratory (Debener et al., 2015; Pacharra et al., 2017).

The data analyzed in the present study source from a large-scale investigation of the driving abilities of older adults (*N* = 395), intended to generate valid indicators and predictors for driving abilities at higher age. The experimental procedure comprised a neuropsychological evaluation, the testing of basic sensory and cognitive abilities as well as the simulated driving test itself. While driving, the EEG was measured in order to generate neurophysiological data, indicating neurocognitive deficits of attentional allocation while driving. In order to fit the whole data acquisition protocol into a reasonable amount of time, especially against the background of the large sample-size, cEEGrid electrodes were chosen as means to record electrophysiological data from the participants. In the first analysis of the EEG data presented here, it was tested, whether task-related modulations of the EEG that are well known from highly controlled laboratory settings, would be as well observable in the EEG recorded in a natural scene driving simulator via cEEGrids.

There is a long history of EEG-measurement in order to analyze the drivers’ mental state. The vast majority of these studies try to detect mental fatigue as a severe risk for accidents on the road (Horne and Reyner, 1999; Horne and Baulk, 2004; Williamson, 2007). Basically, EEG power increases in the lower frequency bands (Delta, Theta, Alpha) with mental fatigue (Cajochen et al., 1995; Lal and Craig, 2001, 2002; Zhao et al., 2012; Arnau et al., 2017). This change in spectral distribution may reflect reduced levels of arousal and alertness (Makeig and Inlow, 1993; Makeig and Jung, 1995; Berka et al., 2005, 2007; Eoh et al., 2005; Tops and Boksem, 2010). Recent studies have shown that Alpha power also changes with task load and task engagement (Wascher et al., 2016). Alpha power was found to be low when participants actively focus on a demanding task (Wascher et al., 2016; Karthaus et al., 2018). On the other hand, very high alpha activity has been reported when mind wandering occurred and participants withdraw their attention from the primary task (Pattyn et al., 2008; Baldwin et al., 2017). More generally, Alpha activity is related to mental states in which errors become more probable (Ogilvie et al., 1991; Jap et al., 2011). Theta power show inverse effects (Wascher et al., 2014; Getzmann et al., 2018). Increased Theta power is in particular observed with higher workload (Wilson and Hankins, 1994; Gevins et al., 1997) or task engagement (Yamada, 1998; Onton et al., 2005). These findings led to different approaches toward EEG-based workload indices (Kumar and Kumar, 2016; Dan and Reiner, 2017; Di Flumeri et al., 2018) that may be applied in order to structure work organization (Chen and Vertegaal, 2004; Huang et al., 2012). Similarly, a number of approaches toward the development of a countermeasure against driver fatigue have been proposed (Lal and Craig, 2001, 2005; Lal et al., 2003; Baldwin et al., 2017), which might be even applied while on-road driving (Papadelis et al., 2009).

Most of the studies mentioned above focus on the general state of the user, measured for longer periods of activity. However, when investigating the spectral properties of the EEG, it is important to differentiate between sustained effects in the ongoing EEG, which reflect primarily the general state of the cognitive system, and task-related spectral modulations, reflecting specific cognitive processes. Also in the analysis of event-related EEG, the modulations of the spectral power in the Theta and the Alpha range were found to be opposed. Theta power usually increases in response to a stimulus, whereas Alpha power decreases (e.g., Onton et al., 2005; Hanslmayr et al., 2012). Moreover, Theta power increases with task complexity (So et al., 2017). The potentially complex interactions between basic state and local demands make general approaches to workload questionable. Therefore, in addition to the general mental states, specific aspects of the information processing requirements in different driving situations must also be investigated. When investigating cognitive processing, data from the laboratory experiment rely on a large number or repetitions of a given event. By averaging EEG segments time locked to this event, random fluctuations and event-related portions of the signal can be separated (Luck, 2005). In realistic environments, the possibility to confront an individual with the same stimulus repeatedly is limited. This holds especially true for driving simulator studies and even more for on-road driving studies, in which an extensive repetition of events is hardly possible. In addition, in realistic contexts it is also hard to control for the occurrence of task-related and task-unrelated cognitive processes that may be present during stimulus presentation. This imposes a great challenge to the analysis of the event-related EEG, as the signal to noise ratio is significantly reduced compared to highly controlled laboratory experiments. This is due to the smaller number of comparable events, as well as to the process-impure nature of the signal recorded. The benefit of sacrificing some internal validity of the obtained measures in e.g., driving simulator scenarios in contrast to laboratory-based tracking tasks, however, is a great improvement of the ecological validity of the study, especially in studies with older participants, who usually benefit from naturalistic settings (Hahn et al., 2013). Prioritizing ecological validity is a necessary step toward the implementation of EEG measures to real-world applications (Ladouce et al., 2017).

Thus, in the present study we evaluated to what extent cEEGrids are suited for EEG measurement in a realistic driving scenario, focusing on the following questions: (1) Is it possible to evaluate factors like mental load by means of cEEGrids? (2) Is it additionally possible to investigate the event-related allocation of attention in specific, singular situations? In order to answer these questions, three different EEG analyses were performed based on the same dataset, all based on a time frequency decomposition of the signal via Morlet wavelet convolution. First, to allow comparison to the vast majority of previous experiments in that field, variations in Alpha and Theta power due to expert-rated task load were analyzed. Second, the time-frequency matrices were transformed into track-frequency structure. Note, since the study intended to observe ecologically valid driving behavior, the variation of average speed was rather high as it is known from older adult drivers (Son et al., 2010; Andrews and Westerman, 2012). Thus, the only systematic way to render the entire track comparable across all participants was to assign the electrophysiological signal to track points rather than to time. Events at particular sections of the route were identical across all participants. Finally, addressing the influences of different driving events, time-frequency responses time locked to a particular moment in time were analyzed.

## Materials and Methods

### Participants

A total of 395 older participants (age range 67–76 years; mean age 71.5 years; SD 2.98 years; 26.6% female) enrolled in the experiment. All participants had a valid driving license and stated to use their car on a regular basis (on average between 5000 and 10000 km/year).

They had normal or corrected to normal vision and reported an overall good health status (97.2%). The most commonly used drugs were hypotensive agents (53.0%), followed by cardiovascular medications (18.6%) and diabetes medications (10.4%).

Before entering the study, all participants provided written informed consent. The study was approved by the local ethics committee of the Leibniz Research Centre for Working Environment and Human Factors.

### Task and Procedure

When entering the laboratory, participants filled out a questionnaire about their driving history, driving habits and attitudes toward driving. After a short test drive to become familiar with the driving simulator, they completed the Montreal Cognitive Assessment (MoCA; Nasreddine et al., 2005) and other neuropsychological tests which will not be reported here. The ride took place in a static driving simulator (ST-Sim, ST Software B.V., Groningen, Netherlands).

The track was designed in a way that it resembled a regular German driving examination with all critical driving situations included. The drive started at a state road with several intersections, roundabouts and a foggy passage before they entered the freeway. There were several roadwork sites to be passed. Back on the state road participants passed several intersections but also drove along a sustained undistorted passage before they entered the city where traffic lights, pedestrians, and cyclists had to be attended. All along the way there was low to moderate traffic.

Participants were guided by navigation signals that indicated upcoming maneuvers. Navigation signals consisted of simultaneously presented acoustic (verbal) and visual information (arrows on an indicator panel near the dashboard). There was no instruction with respect to driving speed. Participants were requested to drive at a comfortable speed as they would go in real traffic in compliance with German road traffic regulations. The distance of the full scenario was 37 km and took the participant between 34 and 85 min (as far as they finished the entire scenario).

### Data Recording and Processing

EEG was recorded with two cEEGrids positioned around the participant’s left and right ear, respectively (see Debener et al., 2015; Bleichner et al., 2016; Mirkovic et al., 2016; Pacharra et al., 2017). The two cEEGrids were connected to a QuickAmp DC-amplifier (Brain Products Inc., Gilching, Germany) with an on-line low pass filtering at 280 Hz. Data were sampled at 1 kHz with a resolution of 24 bits. The two electrodes in the middle of the right cEEGrid served as ground and online reference respectively (R4a, R4b). Data were stored together with base data from the driving simulator (speed, position of the steering wheel).

#### Preprocessing Pipeline

Despite a short phase of familiarization to the simulator (5 to 8 min), there was a substantial drop out due to simulator sickness. 28.6% (*n* = 113) of the participants aborted the experiment before finishing a driven distance of 30 km that was set as the limit for the analyses presented here. This limit was set because participants were offered an exit option thereafter in order to avoid severe simulator sickness evoked by the demanding urban drive. With this distance all types of roads (urban, highway and rural road) were substantially covered. From the remaining 282 participants the first 30 km of the route (which were identical for all participants) were selected for analyses.

EEG data and simulator data were, in a first step, resampled to 200 Hz and then band-pass filtered (1–40 Hz), in particular because slow drifts distort some of the subsequent procedures. Then, single channels of the recorded EEG were checked for integrity by testing both the probability of data as well as their kurtosis by applying the rej_channel function as implemented in EEGLAB (normed data; criterion: 4 standard deviations). Faulty channels were discarded (on average 2.29 channels, SD 2.29; range 0 – 9).

Following the channel rejection procedure, it was checked whether reference channels (L4b, R4b) were still available. 245 data sets remained and were re-referenced to the average of L4b and R4b. These data were entered into the artifact subspace reconstruction (ASR) procedure Mullen et al., 2014, 2017).

Subsequently, a time frequency decomposition was performed on each channel of the remaining data. This was done by convolving the data with complex Morlet wavelets defined as complex sine waves tapered by a Gaussian. We used a set of 29 wavelets with frequencies ranging from 2 to 30 Hz in linearly spaced steps. The widths of the tapering Gaussians were chosen in a way that the spectral resolution was the same at each frequency with a full-width at half-maximum (FWHM; Cohen, 2018) of 2 Hz. This corresponds to a FWHM of 450 ms in the temporal domain. Spectral power estimates were calculated as the squared absolute values of the complex convolution result and averaged across channels.

The obtained data were finally checked for data integrity. Both, subjects with incomplete or corrupt transmission of simulator data into the EEG recording files (*n* = 17) or a total EEG power that deviated by more than three standard deviations from the median (*n* = 12) were discarded. Thus, all following analyses were conducted based on 216 participants.

#### Data Analyses: Estimated Mental Load

In order to test the influences of task load on the driver’s mental status, the driving scenario was classified by an expert according to their assumed mental demand to the driver (task load low, medium, high). Passages of low task load (cumulative 12.83 km) were characterized by an undisturbed ride on a free route; in passages of medium task load (cumulative 8.42 km), junctions with turning, roundabouts, left turns, motorway entrances and exits had to be passed; finally, passages of high task load (cumulative 8.75 km) comprised interactions with other traffic participants, like overtaking, driving behind a vehicle ahead, waiting for oncoming traffic, giving way, avoiding obstacles, construction zone passages, and traffic light intersections (cf. also Pauzié, 2008; Rahman et al., 2017). Task load rating was oriented on specific aspects of functional changes with higher age and their impact on driving behavior (Karthaus and Falkenstein, 2016).

**FIGURE 1:**
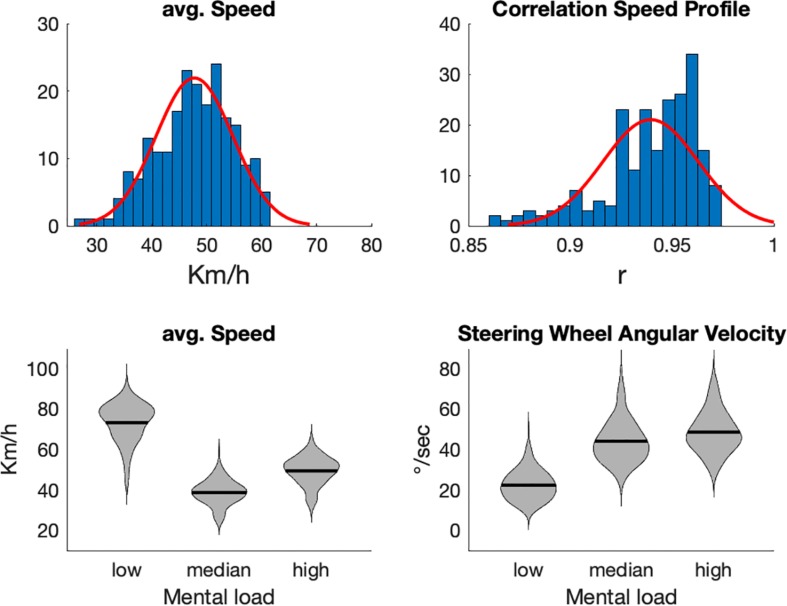
In the upper row distribution of average speed and speed-pattern correlation are shown (the speed profile of each participant was correlated with the grand average of speed profiles). The lower row depicts analyses of driving speed and steering wheel angular velocity with respect to task load (lower row).

**FIGURE 2:**
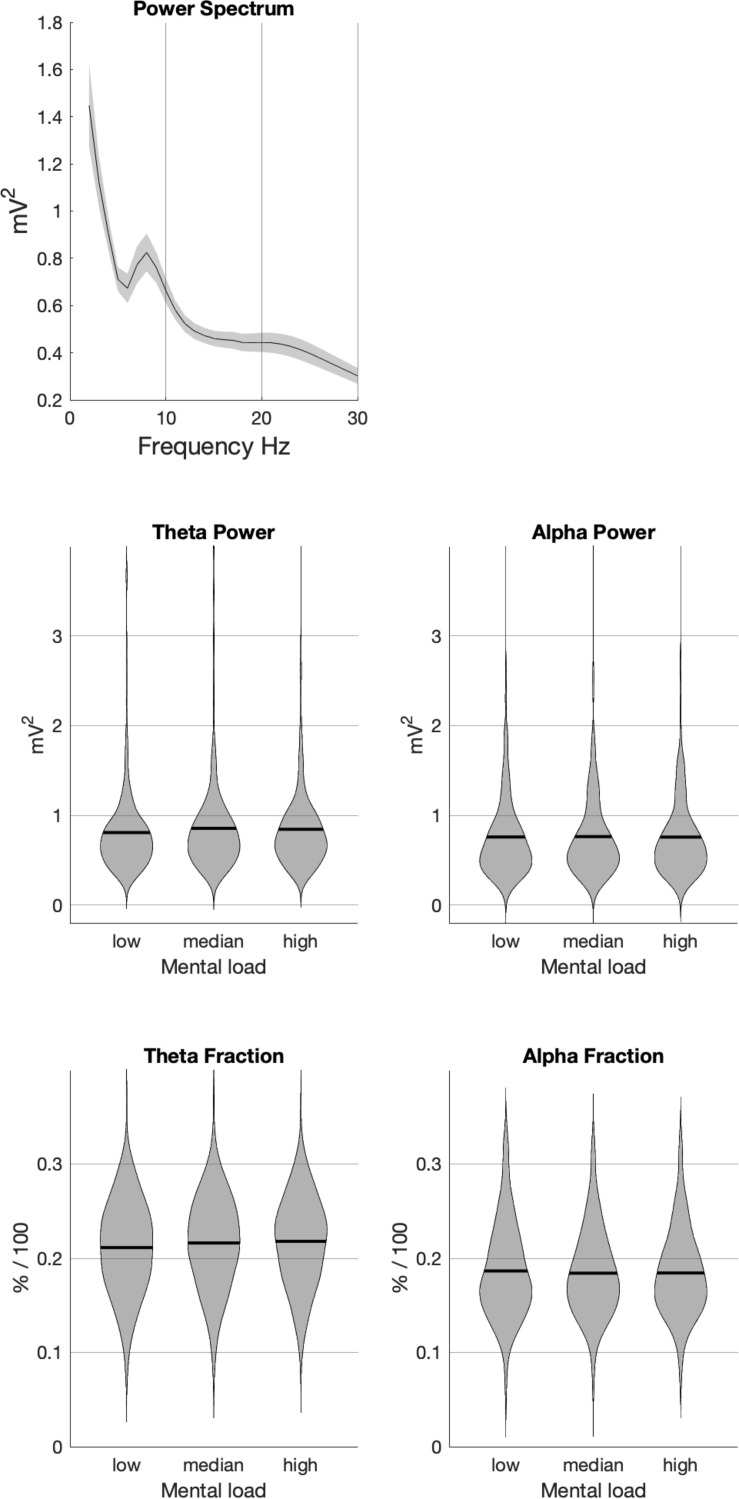
Average power spectrum from all participants **(upper panel)** and analyses of the impact of task load for raw power in the two frequency bands **(middle panel)** and power fractions **(lower panel)**. In the EEG-data, only fractional power revealed strong and reliable effects of task load.

Driving speed, steering wheel angle velocity, and the spectral power in the Theta (3–6 Hz) and the Alpha (7–10 HZ) band were averaged across all segments of each task load and mean values were entered into ANOVAs. Two aspects have to be mentioned here: (1) The speed profiles of participants were highly inter-correlated (see Figure 1). Thus, it can be assumed that each participant despite the high interindividual variance in driving speed spent the same proportion of his/her driving time in either task load condition. (2) The selection of the band borders was slightly shifted to the left compared to traditional analyses because only old adults were tested and at least the individual Alpha frequency has been reported to decrease dramatically with age (Surwillo, 1961; Klimesch, 1999; but see also Figure 2). These frequency borders were kept for all subsequent analyses.

Since at some points of the route massive broadband artifacts (affecting all frequency bands) were still visible despite the extensive pre-processing procedure, we additionally calculated the contribution of each frequency to the overall signal by applying a vector normalization across all frequencies for each time point. These data (called Alpha fraction and Theta fraction) were entered to the same analyses as described for the raw signal above.

#### Data Analyses: The Track-Frequency Analysis

The track-based analysis bases on the knowledge of 43 predefined landmarks that were set during EEG recording. For each participant, a TTL-trigger was set in the EEG recording file when the vehicle passed these points. In-between two landmarks, for each time point of recording, the waypoint on track was estimated based on vehicle speed as transmitted from the simulator. The estimate included the upcoming landmark and was compared to the known position of this point. The maximal error for all estimates was below 1.5%.

In a second step, segments were built 10-meter wise and mean power fraction was calculated for each frequency band for each segment. The obtained data were *z*-transformed across all 3000 data points and low-pass filtered by an ± 40 m moving average. The 95% confidence interval was calculated and depicted in Figure 2.

Based on this rather descriptive analysis, a simple algorithm is proposed to classify task load based on EEG data. Starting with the assumption that high Theta activity is related to mental effort whereas high Alpha is related to reduced attentional allocation, for each time point Theta and Alpha fraction were tested against each other by a paired sample *t*-test. Significantly higher Alpha fraction was assigned to low task load, increased Theta as high task load. Waypoints with no differences between the two frequency bands were assigned to median task load. This classification was correlated with the expert rating.

Strong differences between the two classification approaches (expert vs. EEG) will be discussed based on properties of the respective road section.

## Results

### Behavior

Average speed (see Figure 1, upper left graph) varied markedly across participants, however, showed a good approximation to a normal distribution. Mean driving speed was 47.79 km/h. The variations across participants (standard deviation = 6.99 km/h) reflected the well-known strategy of older adults to reduce speed when they feel unsecure (Trick et al., 2010).

When mental load is considered as derived from an expert rating, driving parameters (see Figure 1 lower row) fit these estimates. Speed varied reliably with task load, *F*(2,215) = 5356.5, *p* < 0.001. Decrease in speed was in particular pronounced from low to median load, *F*(1,215) = 7284.5, *p* < 0.001. From median to high task load, driving speed increased again, *F*(1,215) = 1347.9, *p* < 0.001.

Steering wheel angular velocity also varied with task load, *F*(2,215) = 3040.3, *p* < 0.001. It increased steadily from low to median, *F*(1,215) = 3240, *p* < 0.001, and from median to high task load, *F*(1,215) = 212.15, *p* < 0.001.

Thus, as defined in the criteria of the expert rating, straight and undistorted driving defined low task load. Medium and high task load showed comparable driving speeds, however with medium task load showing a reduced steering activity compared to high task load.

### Spectral Analyses

The power spectra obtained (see Figure 2, first row) nicely reflect an 1/f function. The only distinct peak in the spectra most probably reflects Alpha activity. Although its maximum was below 9 Hz, the high age of participants would predict such slow Alpha activity (Klimesch, 1999) and therefore support the validity of data.

Raw Theta power varied with task load, *F*(2,215) = 5.982, *p* = 0.002. The power increased from low to median task load, *F*(1,215) = 20.082, *p* < 0.001, but did not differ between median and high, *F*(1,215) = 0.362, *p* > 0.2. Raw Alpha power did not change with task load, *F*(1,215) = 0.541, *p* > 0.2.

More differentiated was the picture obtained with power fractions. Theta fraction varied with task load, *F*(2,215) = 82.167, *p* < 0.001. Theta fraction increased from low to median, *F*(1,215) = 69.442, *p* < 0.001, and from median to high task load, *F*(1,215) = 11.435, *p* < 0.001. Also Alpha fraction showed a significant effect, *F*(2,215) = 8.9843, *p* < 0.001. It decreased from low to median task load, *F*(1,215) = 12.181, *p* < 0.001, but no further when median and high task load are compared, *F*(1,215) = 0.243, *p* > 0.2.

### The Track-Frequency Analysis

**FIGURE 3:**
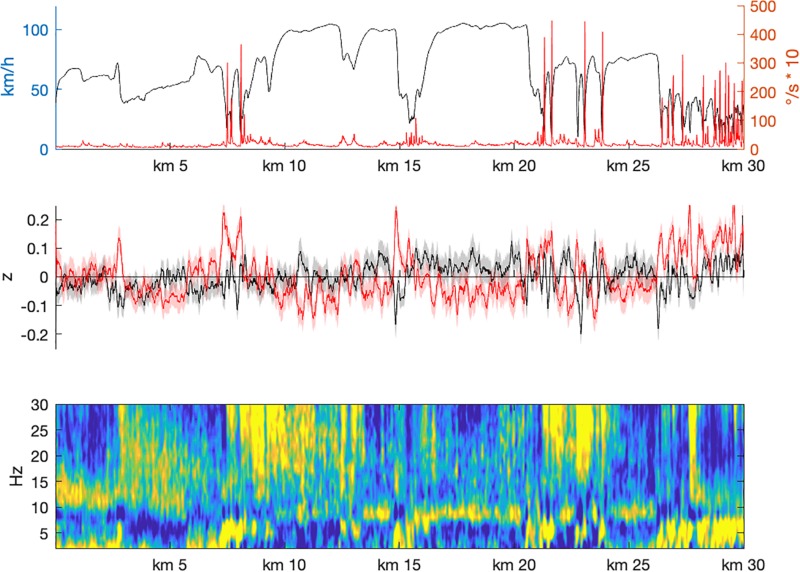
Track-based analyses of driving parameter (**upper panel**; black: driving speed; red: steering wheel angular velocity) and EEG-data. Note that each time point of each participant was assigned to a waypoint. All measures presented here were then averaged 10-meter wise. The **middle panel** shows mean Alpha (black) and Theta (red) fractions shaded by their 95% confidence interval. The lower panel depicts the entire spectrum.

The track-frequency analysis (see Figure 3) refers to average values of each parameter for 10 m segments of the scene. It supports the findings from the mental load analysis. Segments with high Theta fractions are characterized by increased steering wheel angular velocity due to junctions or roundabouts. This is in particular visible when participants entered the city (around 27 km). High Alpha fraction was only visible in segments where participants drove on a more or less constant speed without much steering activity. As indicated already from the rating-based task load analysis, Alpha suppression did not take place on a constant level for a longer time period, which might explain the missing effect on Alpha fraction with high task load.

**FIGURE 4:**
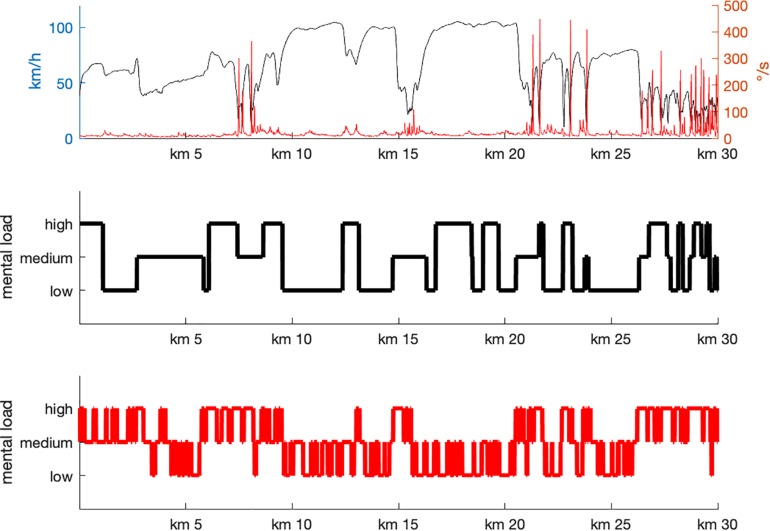
Track-based driving parameter and estimates of task load as derived from an expert rating **(middle panel)** and the track-frequency analysis **(lower panel)**. There are substantial similarities between the two classifications, but also strong differences e.g., when participants entered the foggy passage (around 3 km), on the freeway (between 17 and 20 km) and when entering the city (27 km).

In a second step, an estimate of task load was derived based on the track-frequency analysis. Segments in which Theta was significantly higher than Alpha fraction were denoted as high task load. The reversed effect was assigned to low task load. Segments where no difference between the two frequency bands were found were assigned to medium task load (see Figure 4).

The task load values from the expert rating and the calculated estimates of task load correlated with *r* = 0.311, *p* < 0.001, indicating that there was some similarity between the two measures, however, that there were as well some substantial differences.

Looking at the most prominent differences includes the following driving segments:

(1) Driving into the foggy passage was not assigned to medium mental load, as proposed by the expert, but showed a short passage of high task load that rapidly declined to low task load in the track-frequency analysis.
(2) Driving on the freeway was not demanding when interaction with other road users was required.
(3) Driving into the city was highly demanding for the participants independent of the specific requirements in a particular situation.

**FIGURE 5:**
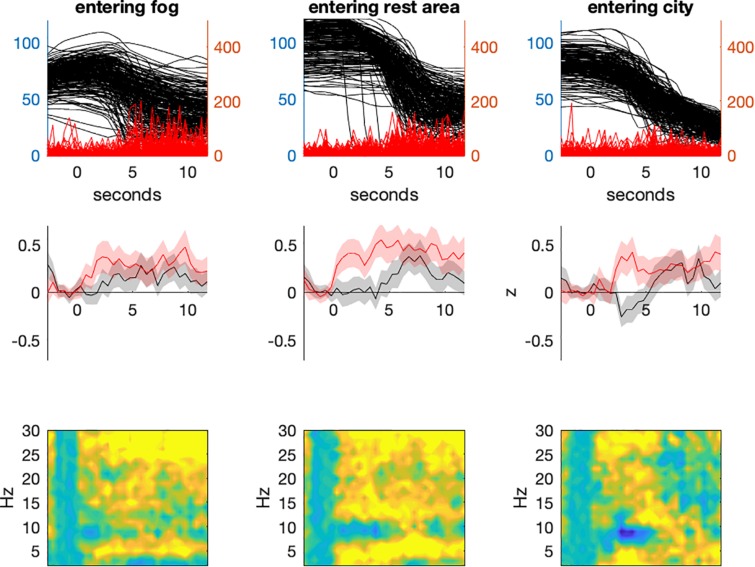
Time frequency based analyses of entering new situations. When entering the foggy passage **(left panel)**, the rest area on the freeway **(middle panel)** or the first junction in the city **(right panel)**. A short and phasic increase in Theta activity can be observed but not in the Alpha band.

Apart from these global aspects, it is to be mentioned that high task load appeared to be a rather local phenomenon, occurring primarily when participants entered a new situation. In order to demonstrate these situations, three positions were selected where participants had to break and entered a demanding segment (see Figure 5). In all cases a short, phasic increase in Theta fraction is visible accompanied by low Alpha activity.

## Discussion

The present study intended to investigate neurophysiological parameters of mental load in older adults while driving along a realistic scene in a driving simulator. Due to temporal constraints in the experimental design no full-cap EEG was recorded, but film-based round the cEEGrids were used to pick up the EEG signal. Here we demonstrate how reliable and valid the obtained EEG signal from this type of electrodes was and to what degree the EEG data reflect mental load.

Participants drove a scenario that included all elements of a regular German driving test (except parking). They were not instructed to drive at a specific speed, which might explain the high variability in this parameter. One of the main compensation mechanisms with respect to driving behavior in older adults is an adaptation of driving speed when they feel unsecure (Trick et al., 2010; Andrews and Westerman, 2012). Thus, the data pattern measured in this experimental setting is well in line with natural driving behavior.

Raw inspection of the data revealed segments with large motion artifacts that affected the entire frequency range. Here it is to be mentioned that film-based electrodes on the skin of older participants did not always reveal a good signal. Regular aging of the skin leads to wrinkles that prevented even adhesion of the film electrodes. It appears plausible that this fact amplified the distortion of the signal due to motion artifacts. However, applying Artifact Subspace Reconstruction, revealed a stable signal that showed a well-defined and plausible measure for almost all participants.

The first analysis of data was based on an expert rating that was guided by known factors of mental load in driving situations (Pauzié, 2008; Engström et al., 2017; Rahman et al., 2017) and by known critical issues of older drivers due to functional decline (Karthaus and Falkenstein, 2016). The driving parkours was *a priori* subdivided in passages of low, medium, and high task load, characterized by an undisturbed ride on a free route, passages with junctions with turning, roundabouts, and left turns, as well as interactions with other traffic participants, respectively. One has to be aware, however, that in particular in older adults the variance of driving strategies and perceived task load are highly variable (Andrews and Westerman, 2012). Nevertheless, the criteria applied appeared to be a suitable first guess for differences in task demands that are also reflected in the behavioral data. Driving speed and steering activity varied plausibly with task load.

Applying this categorization to EEG data revealed results that are well in accordance with the literature. Aspects of Alpha and Theta activity varied with task load. For the raw power, a modulation with task load was only visible in the Theta band. Relative power (power fraction), however, varied in the Alpha and in the Theta band in the expected direction. Theta increased with task load and Alpha decreased (Wascher et al., 2016). Such a discrepancy between raw and relative power is not unusual (Arnau et al., 2017). In particular the high variability of raw power (as also visible in Figure 2) might prevent effects from becoming significant.

On the other hand, it is also likely that the expert rating did not fully coincides with the demands as they are perceived by the older population that was tested. In particular the interaction with other road users that is generally assumed to be demanding (Rahman et al., 2017), might result in a less demanding situation exerting very defensive driving behavior. This is usually attributed to compensation strategies deployed by older drivers (e.g., Karthaus and Falkenstein, 2016) to avoid i.e., overtaking maneuvers.

In order to challenge these shortcomings, an approach was used that might provide EEG-based workload indices (Kumar and Kumar, 2016; Dan and Reiner, 2017; Di Flumeri et al., 2018) for every section of the track in high spatial resolution. Each time point of the individual EEG recording of each participant was assigned to a particular waypoint by stretching and compressing the data in the temporal domain. Based on this assignment, spectral power estimates were averaged for 10-meter segments. Then, Alpha and Theta fractions were calculated for each of these segments. The pattern of the course of these two frequency traces varied systematically with the characteristics of the assigned street segments. Reliable high Theta activity was only visible at very prominent loci where much activity and attentional allocation were required. In contrast, Alpha activity was high when participants drove undistorted on free street segments, without much activity.

Thus, an estimate for a workload index was calculated based on Alpha and Theta power and subsequently compared to the expert rating. There was a substantial correlation between the two indices but also remarkable deviations. These deviations, however, might appear to be plausible when analyzing the situation more closely, in which they occurred.

The first situation was the foggy passage. The expert rated this situation as medium task load, because all the way vision was disturbed, but sufficient for save driving at reduced speed. Therefore, a higher amount of attention was expected to be required, relative to driving in conditions with good visibility conditions. In fact, the EEG-based estimation of workload was high when entering the fog passage. While driving through the fog, the EEG pattern suggested low task load. Looking at the behavior, it became clear that participants did not behave as initially expected. At the beginning of the fog passage, they strongly reduced driving speed, but then continuously accelerated despite the profoundly reduced visibility. It is a well-known phenomenon, however, that driving speed may be heavily misperceived in fog (Brooks et al., 2011; Mueller and Trick, 2012), which is one of the reasons for severe accidents. Obvious risks might be ignored and lead to reduced perceived task load, as also found here in older adults. It is to be mentioned that passing a junction in the fog (after about 4 km drive) led to a short reduction in speed and a short moment of higher task load (cf., Figure 3). More generally, approaching a new situation went along with phasic increases of task load in measured EEG. The longest period of deviating estimates of task load was a passage on the freeway with increased traffic and therefore increased interaction with other road users. The assumption of the expert that interaction might lead to increased task load turned out to be wrong. The possibility to flexibly adjust behavior in such a situation (by reducing the driving speed, for example) might have rendered the situation less demanding. Finally, entering the city revealed EEG-based task load estimates that were always at a medium or high level. Obviously, the rapid sequence of events in urban traffic prevented the drivers’ attentional system from going into a relaxed state of low task load. Thus, there are plausible explanations for the deviations between the expert rating and the EEG-based estimates which are in fact in favor of the latter.

One possible flaw remains at this point. The EEG-based estimate of task load might be driven by motor rather than cognitive demands. Activity in the Alpha frequency range is also closely related to motor activity. So-called Mu-activity (8–12 Hz over sensorimotor areas) is suppressed in relation to motor processes and sensory motor integration (for a review see Pineda, 2005). Because the data quality of cEEGrids did not allow source allocation of the obtained signal, such activity cannot be dissociated from Alpha activity. However, there are two arguments against this notion: (1) as can be seen in Figure 5, the strongest cognitive demands when entering the city came along with the lowest steering (motor) activity. (2) A *post hoc* correlation between task load and steering activity revealed a rather week correlation (*r* = 0.132).

In summary, it was demonstrated that cEEGrids did not only provide a reliable EEG signal but also data that allowed for a valid interpretation of task load in a minimally controlled, realistic task. For sure, task load was not well controlled in the driving task but it was approached under high ecological validity. Derived from an activity in the Alpha and the Theta band, an estimate of task demands was generated that at least *a posteriori*, in a detailed analysis, appeared to explain task load of a driving situation more plausible than a criterion-based rating of an expert. The findings presented here therefore create new opportunities in cognitive neuro-ergonomics and mobility research. Since the system is in principle completely mobile, real-world applications become possible and might generate access to an objective measure of task load in any context.

## Ethics Statement

The study was approved by Ethik committee of the IfADo.

## Author Contributions

EW, GRu, MK, GRi, FD, and SG designed study. JR acquired the data and provided the data and analyses of a pilot-study. SA evaluated and programing of data analysis tools. GRi and FD provided additional analysis approaches and methods. EW wrote the first version of the manuscript. All authors commented on and provided valuable comments for the final version of the manuscript.

## Conflict of Interest Statement

The authors declare that the research was conducted in the absence of any commercial or financial relationships that could be construed as a potential conflict of interest.

## References

[B1] Andrews E. C. Westerman S. J. (2012). Age differences in simulated driving performance: compensatory processes. *Accid. Anal. Prev.* 45 660–668. 10.1016/j.aap.2011.09.047 22269555

[B2] Arnau S. Möckel T. Rinkenauer G. Wascher E. (2017). The interconnection of mental fatigue and aging_ An EEG study. *Int. J. Psychophysiol.* 117 17–25. 10.1016/j.ijpsycho.2017.04.003 28400244

[B3] Baldwin C. L. Roberts D. M. Barragan D. Lee J. D. Lerner N. Higgins J. S. (2017). Detecting and quantifying mind wandering during simulated driving. *Front. Hum. Neurosci.* 11:406. 10.3389/fnhum.2017.00406 28848414 PMC5550411

[B4] Berka C. Levendowski D. J. Lumicao M. N. Yau A. Davis G. Zivkovic V. T. (2007). EEG correlates of task engagement and mental workload in vigilance, learning, and memory tasks. *Aviat. Space Environ. Med.* 78(5 Suppl.), B231–B244. 17547324

[B5] Berka C. Levendowski D. J. Westbrook P. Davis G. Lumicao M. N. Olmstead R. E. (2005). “EEG quantification of alertness: methods for early identification of individuals most susceptible to sleep deprivation,” in *Biomonitoring for Physiological and Cognitive Performance during Military Operations*, Vol. 5797 eds Caldwell J. A. Wesensten N. J. (Orlando, FL: International Society for Optics and Photonics), 78–90.

[B6] Bleichner M. G. Debener S. (2017). Concealed, unobtrusive ear-centered EEG acquisition: cEEGrids for transparnt EEG. *Front. Hum. Neurosci.* 11:163. 10.3389/fnhum.2017.00163 28439233 PMC5383730

[B7] Bleichner M. G. Mirkovic B. Debener S. (2016). Identifying auditory attention with ear-EEG: cEEGrid versus high-density cap-EEG comparison. *J. Neural Eng.* 13:066004. 10.1088/1741-2560/13/6/066004 27705963

[B8] Brooks J. O. Crisler M. C. Klein N. Goodenough R. Beeco R. W. Guirl C. (2011). Speed choice and driving performance in simulated foggy conditions. *Accid. Anal. Prev.* 43 698–705. 10.1016/j.aap.2010.10.014 21376857

[B9] Cajochen C. Brunner D. P. Krauchi K. Graw P. Wirz-Justice A. (1995). Power density in theta/alpha frequencies of the waking EEG progressively increases during sustained wakefulness. *Sleep* 18 890–894. 10.1093/sleep/18.10.890 8746397

[B10] Chen D. Vertegaal R. (2004). “Using mental load for managing interruptions in physiologically attentive user interfaces,” in *Proceedings of the Extended abstracts of the 2004 Conference on Human Factors in Computing Systems, CHI 2004*, Vienna, 1513–1516.

[B11] Cohen M. X. (2018). A better way to define and describe Morlet wavelets for time-frequency analysis. *BioRxiv* 397182. 10.1016/j.neuroimage.2019.05.048 31145982

[B12] Dan A. Reiner M. (2017). Real time EEG based measurements of cognitive load indicates mental states during learning. *J. Educ. Data Mining* 9 31–44.

[B13] Debener S. Emkes R. De Vos M. Bleichner M. (2015). Unobtrusive ambulatory EEG using a smartphone and flexible printed electrodes around the ear. *Sci. Rep.* 5:16743. 10.1038/srep16743 26572314 PMC4648079

[B14] Denk F. Grzybowski M. Ernst S. M. A. Kollmeier B. Debener S. Bleichner M. G. (2018). Event-related potentials measured from in and around the ear electrodes integrated in a live hearing device for monitoring sound perception. *Trends Hear.* 22:2331216518788219. 10.1177/2331216518788219 30022733 PMC6053864

[B15] Di Flumeri G. Borghini G. Aricò P. Sciaraffa N. Lanzi P. Pozzi S. (2018). EEG-based mental workload neurometric to evaluate the impact of different traffic and road conditions in real driving settings. *Front. Hum. Neurosci.* 12:509. 10.3389/fnhum.2018.00509 30618686 PMC6305466

[B16] Engström J. Markkula G. Victor T. Merat N. (2017). Effects of cognitive load on driving performance: the cognitive control hypothesis. *Hum. Factors* 59 734–764. 10.1177/0018720817690639 28186421

[B17] Eoh H. J. Chung M. K. Kim S.-H. (2005). Electroencephalographic study of drowsiness in simulated driving with sleep deprivation. *Int. J. Ind. Ergon.* 35 307–320. 10.1016/j.ergon.2004.09.006

[B18] Getzmann S. Arnau S. Karthaus M. Reiser J. E. Wascher E. (2018). Age-related differences in pro-active driving behavior revealed by EEG measures. *Front. Hum. Neurosci.* 12:321. 10.3389/fnhum.2018.00321 30131687 PMC6090568

[B19] Gevins A. Smith M. E. McEvoy L. Yu D. (1997). High-resolution EEG mapping of cortical activation related to working memory: effects of task difficulty, type of processing, and practice. *Cereb. Cortex* 7 374–385. 10.1093/cercor/7.4.374 9177767

[B20] Hahn M. Wild-Wall N. Falkenstein M. (2013). “Age-related changes of neural control processes and their significance for driving performance,” in *Age-Differentiated Work Systems*, eds Schlick C. M. Frieling E. Wegge J. (Heidelberg: Springer), 299–317. 10.1007/978-3-642-35057-3_13

[B21] Hanslmayr S. Staudigl T. Fellner M.-C. (2012). Oscillatory power decreases and long-term memory: the information via desynchronization hypothesis. *Front. Hum. Neurosci.* 3:74. 10.3389/fnhum.2012.00074 22514527 PMC3322486

[B22] Horne J. Reyner L. (1999). Vehicle accidents related to sleep: a review. *Occup. Environ. Med.* 56 289–294. 10.1136/oem.56.5.289 10472301 PMC1757738

[B23] Horne J. A. Baulk S. D. (2004). Awareness of sleepiness when driving. *Psychophysiology* 41 161–165. 10.1046/j.1469-8986.2003.00130.x 14693012

[B24] Huang K.-C. Jung T.-P. Chuang C.-H. Ko L.-W. Lin C.-T. (2012). Preventing lapse in performance using a drowsiness monitoring and management system. *Conf. Proc. IEEE Eng. Med. Biol. Soc.* 2012 3336–3339. 10.1109/EMBC.2012.6346679 23366640

[B25] Jap B. T. Lal S. Fischer P. (2011). Comparing combinations of EEG activity in train drivers during monotonous driving. *Expert Syst. Appl.* 38 996–1003. 10.1016/j.eswa.2010.07.109

[B26] Karthaus M. Falkenstein M. (2016). Functional changes and driving performance in older drivers: assessment and interventions. *Geriatrics* 1:12. 10.3390/geriatrics1020012 31022806 PMC6371115

[B27] Karthaus M. Wascher E. Getzmann S. (2018). Proactive vs. reactive car driving: EEG evidence for different driving strategies of older drivers. *PLoS One* 13:e191500. 10.1371/journal.pone.0191500 29352314 PMC5774811

[B28] Klimesch W. (1999). EEG alpha and theta oscillations reflect cognitive and memory performance: a review and analysis. *Brain Res. Brain Res. Rev.* 29 169–195. 10.1016/s0165-0173(98)00056-3 10209231

[B29] Kumar N. Kumar J. (2016). Measurement of cognitive load in HCI systems using EEG power spectrum: an experimental study. *Proc. Comput. Sci.* 84 70–78. 10.1016/j.procs.2016.04.068

[B30] Ladouce S. Donaldson D. I. Dudchenko P. A. Ietswaart M. (2017). Understanding minds in real-world environments: toward a mobile cognition approach. *Front. Hum. Neurosci.* 10:696. 10.3389/fnhum.2016.00694 28127283 PMC5226959

[B31] Lal S. K. L. Craig A. (2001). A critical review of the psychophysiology of driver fatigue. *Biol. Psychol.* 55 173–194. 10.1016/s0301-0511(00)00085-5 11240213

[B32] Lal S. K. L. Craig A. (2002). Driver fatigue: electroencephalography and psychological assessment. *Psychophysiology* 39 313–321. 10.1017/s0048577201393095 12212650

[B33] Lal S. K. L. Craig A. (2005). Reproducibility of the spectral components of the electroencephalogram during driver fatigue. *Int. J. Psychophysiol.* 55 137–143. 10.1016/j.ijpsycho.2004.07.001 15649545

[B34] Lal S. K. L. Craig A. Boord P. Kirkup L. Nguyen H. (2003). Development of an algorithm for an EEG-based driver fatigue countermeasure. *J. Safety Res.* 34 321–328. 10.1016/s0022-4375(03)00027-6 12963079

[B35] Luck S. J. (2005). *An Introduction to the Event-Related Potential Technique.* Cambridge: MIT Press.

[B36] Makeig S. Inlow M. (1993). Lapses in alertness: coherence of fluctuations in performance and EEG spectrum. *Electroencephalogr. Clin. Neurophysiol.* 86 23–35. 10.1016/0013-4694(93)90064-3 7678388

[B37] Makeig S. Jung T. (1995). Changes in alertness are a principal component of variance in the EEG spectrum. *Neuroreport* 7 213–216. 10.1097/00001756-199512290-00051 8742454

[B38] Mikkelsen K. B. Ebajemito J. K. Bonmati-Carrion M. A. Santhi N. Revell V. L. Atzori G. (2019). Machine-learning-derived sleep–wake staging from around-the-ear electroencephalogram outperforms manual scoring and actigraphy. *J. Sleep Res.* 28:e12786. 10.1111/jsr.12786 30421469 PMC6446944

[B39] Mirkovic B. Bleichner M. G. De Vos M. Debener S. (2016). Target speaker detection with concealed EEG around the ear. *Front. Neurosci.* 10:349. 10.3389/fnins.2016.00349 27512364 PMC4961688

[B40] Mueller A. S. Trick L. M. (2012). Driving in fog: the effects of driving experience and visibility on speed compensation and hazard avoidance. *Accid. Anal. Prev.* 48 472–479. 10.1016/j.aap.2012.03.003 22664714

[B41] Mullen T. Kothe C. Chi Y. M. Ojeda A. Kerth T. Makeig S. (2014). “Real-time modeling and 3D visualization of source dynamics and connectivity using wearable EEG,” in *Presented at the 2013 35th Annual International Conference of the IEEE Engineering in Medicine and Biology Society (EMBC)*, (Piscataway, NJ: IEEE), 2184–2187. 10.1109/EMBC.2013.6609968 PMC4119601 24110155

[B42] Mullen T. R. Kothe C. A. E. Chi Y. M. Ojeda A. Kerth T. Makeig S. (2017). Real-time neuroimaging and cognitive monitoring using wearable dry EEG. *IEEE Trans. Biomed. Eng.* 62 2553–2567. 10.1109/tbme.2015.2481482 26415149 PMC4710679

[B43] Nasreddine Z. S. Phillips N. A. Bédirian V. Charbonneau S. Whitehead V. Collin I. (2005). The montreal cognitive assessment, MoCA: a brief screening tool for mild cognitive impairment. *J. Am. Geriatr. Soc.* 53 695–699. 10.1111/j.1532-5415.2005.53221.x 15817019

[B44] Ogilvie R. D. Simons I. A. Kuderian R. H. MacDonald T. Rustenburg J. (1991). Behavioral, event-related potential, and EEG/FFT changes at sleep onset. *Psychophysiology* 28 54–64. 10.1111/j.1469-8986.1991.tb03386.x 1886964

[B45] Onton J. Delorme A. Makeig S. (2005). Frontal midline EEG dynamics during working memory. *Neuroimage* 27 341–356. 10.1016/j.neuroimage.2005.04.014 15927487

[B46] Pacharra M. Debener S. Wascher E. (2017). Concealed around-the-ear EEG captures cognitive processing in a visual Simon task. *Front. Hum. Neurosci.* 11:290. 10.3389/fnhum.2017.00290 28642695 PMC5462961

[B47] Papadelis C. L. Lithari C. Kourthidou-Papadeli C. Bamidis P. D. Portouli E. Bekiaris E. (2009). “Monitoring driver’s sleepiness on-board for preventing road accidents,” in *Medical Informaticy in a United and Healthy Europe*, ed. Adlassnig K. P. (Amsterdam: IOS Press), 485–489. 19745359

[B48] Pattyn N. Neyt X. Henderickx D. Soetens E. (2008). Psychophysiological investigation of vigilance decrement: boredom or cognitive fatigue? *Physiol. Behav.* 93 369–378. 10.1016/j.physbeh.2007.09.016 17999934

[B49] Pauzié A. (2008). A method to assess the driver mental workload: the driving activity load index (DALI). *IET Intell. Trans. Syst.* 2 315–319.

[B50] Pineda J. A. (2005). The functional significance of mu rhythms: translating “seeing” and “hearing” into “doing”. *Brain Res. Rev.* 50 57–68. 10.1016/j.brainresrev.2005.04.005 15925412

[B51] Rahman N. I. A. Dawal S. Z. M. Yusoff N. (2017). Subjective responses of mental workload during real time driving: a pilot field study. *IOP Conf. Ser. Mater. Sci. Eng.* 210 12076–12079.

[B52] So W. K. Y. Wong S. W. H. Mak J. N. Chan R. H. M. (2017). An evaluation of mental workload with frontal EEG. *PLoS One* 12:e174949. 10.1371/journal.pone.0174949 28414729 PMC5393562

[B53] Son J. Reimer B. Mehler B. Pohlmeyer A. E. Godfrey K. M. Orszulak J. (2010). Age and cross-cultural comparison of drivers’ cognitive workload and performance in simulated urban driving. *Int. J. Automot. Technol.* 11 533–539. 10.1007/s12239-010-0065-6

[B54] Sterr A. Ebajemito J. K. Mikkelsen K. B. Bonmati-Carrion M. A. Santhi N. Della Monica C. (2018). Sleep EEG derived from behind-the-ear electrodes (cEEGrid) compared to standard polysomnography: a proof of concept study. *Front. Hum. Neurosci.* 12:452. 10.3389/fnhum.2018.00452 30534063 PMC6276915

[B55] Surwillo W. W. (1961). Frequency of the “Alpha” rythm, reaction time and age. *Nature* 191 823–824. 10.1038/191823a0

[B56] Symeonidou E.-R. Nordin A. Hairston W. Ferris D. (2018). Effects of cable sway, electrode surface area, and electrode mass on electroencephalography signal quality during motion. *Sensors* 18 1–13. 10.3390/s18041073 29614020 PMC5948545

[B57] Tops M. Boksem M. A. S. (2010). Absorbed in the task: personality measures predict engagement during task performance as tracked by error negativity and asymmetrical frontal activity. *Cogn. Affect. Behav. Neurosci.* 10 441–453. 10.3758/CABN.10.4.441 21098805

[B58] Trick L. M. Toxopeus R. Wilson D. (2010). The effects of visibility conditions, traffic density, and navigational challenge on speed compensation and driving performance in older adults. *Accid. Anal. Prev.* 42 1661–1671. 10.1016/j.aap.2010.04.005 20728615

[B59] Wascher E. Getzmann S. Karthaus M. (2016). Driver state examination—Treading new paths. *Accid. Anal. Prev.* 91 157–165. 10.1016/j.aap.2016.02.029 26986022

[B60] Wascher E. Rasch B. Sänger J. Hoffmann S. Schneider D. Rinkenauer G. (2014). Frontal Theta activity reflects distinct aspects of mental fatigue. *Biol. Psychol.* 96 57–65. 10.1016/j.biopsycho.2013.11.010 24309160

[B61] Williamson A. (2007). “Fatigue and coping with driver distraction,” in *Distracted Driving*, eds Faulks I. J. Regan M. Stevenson M. Brown J. Porter A. Irwin J. D. (Sydney, NSW: Australasian College of Road Safety), 611–622.

[B62] Wilson G. F. Hankins T. (1994). Eeg and subjective measures of private pilot workload. *Proc. Hum. Factors Ergon. Soc. Annu. Meet.* 38 1322–1325. 10.1177/154193129403801916

[B63] Yamada F. (1998). Frontal midline theta rhythm and eyeblinking activity during a VDT task and a video game: useful tools for psychophysiology in ergonomics. *Ergonomics* 41 678–688. 10.1080/001401398186847 9613228

[B64] Zhao C. Zhao M. Liu J. Zheng C. (2012). Electroencephalogram and electrocardiograph assessment of mental fatigue in a driving simulator. *Accid. Anal. Prev.* 45 83–90. 10.1016/j.aap.2011.11.019 22269488

